# Impacts of experimental advisory exit speed sign on traffic speeds for freeway exit ramp

**DOI:** 10.1371/journal.pone.0225203

**Published:** 2019-11-20

**Authors:** Yongfeng Ma, Wenbo Zhang, Xin Gu, Jiguang Zhao

**Affiliations:** 1 School of Transportation, Southeast University, Nanjing, Jiangsu, China; 2 Jiangsu Key Laboratory of Urban ITS, Nanjing, Jiangsu, China; 3 Jiangsu Collaborative Innovation Center of Modern Urban Traffic Technologies, Nanjing, Jiangsu, China; 4 HNTB Corporation, Tallahassee, FL, United States of America; Tongii University, CHINA

## Abstract

Many crashes occur around freeway exit ramp areas in China due to excessive speeds and large speed variances. Traditionally, a single posted ramp speed limit sign is installed around the physical gore area to manage the speed. To address this issue, the study presented in this paper proposes the use of an advisory exit speed sign (AESS), which is an additional exit speed limit sign positioned along the deceleration lane to accommodate the speed changes ahead of the physical gore. The study selected three sites with similar exit ramp configurations and two scenarios (with AESS/without AESS) to quantify the influences of the AESS on the speed of exiting vehicles. The speed profiles of 480 vehicles were obtained based on 12 hours of data collection. A t-test was applied to verify the reduction in mean speed between the two scenarios. The results show that the AESS in this study was effective in reducing the mean speed and 85th percentile speed, especially in the taper and deceleration lane. It was clearly seen that drivers began to decelerate in advance when the AESS was installed, which led to a smooth deceleration process, especially on the segment between the theoretical gore and the physical gore. The AESS was also helpful in reducing speeding to some extent. Although the effects of the AESS on speed reduction at curved ramps were not ideal, the speed fluctuation range tended to be more contracted when the AESS was installed. This paper provides useful information for researchers, managers, and engineers when considering the implementation of AESS.

## Introduction

The exit ramp is considered the most dangerous area on a freeway compared to other freeway segments. Drivers’ workload and errors increase significant at diverging areas [1–4]. Evidence show that more than 80% freeway crashes were found to occur at ramp terminals, and the number of crashes occurring at freeway exit ramps was twice the number of crashes that occurred at entrance ramp terminals [5–10]. Vehicle rollover and rear-end crashes are the most frequently observed crash types at freeway exit ramps, because drivers need to decelerate and change lane in a limited space. Contributing factors for the high crash frequency at exit ramp areas include speeding and greater speed variance [11–15]. Guo et al. concluded that speed limit, and speed difference present significant heterogeneous effects on crash rates at freeway diverge areas [12,13]. The speeds at exit ramps are generally greater than the posted ramp speed limit by 7 to 10 miles per hour (mph) [16]. Data collected from freeway exit ramps in Houston and Texas indicated that all types of vehicles exceeded the posted speed limits by 5 to 15 mph, and in some cases, more than 15 mph [17]. Many studies have been conducted to explore the impact of the speed difference diversity on driver behaviors based on field or simulator data, also their consequences for traffic operation and safety at freeway diverging areas [17–26].

To address safety and operation issues, some speed control techniques have been designed to manage speed at exit ramp areas(e.g., conventional signs, markings, and rubber speed humps)[27–34]. For example, Retting et al found that pavement markings were generally effective in reducing the speeds of passenger vehicles and large trucks at four exit ramps [27]. Freedman et al. examined alternative signage for freeway exit ramps at risk for rollover crashes, and concluded that the flashing sign, which activated for trucks that tend to exceed the advisory speed was more effective than a non-flashing advisory speed sign [28]. Reddy et al. evaluated the effectiveness of the Tyregrip high friction surface and found that drivers tended to slow down when traveling through the ramp areas treated with the Tyregrip surface [29]. Hunter et al. conducted a before-after study to investigate the effectiveness of chevron markings on reducing vehicle speeds around ramp areas in Atlanta, Georgia[30]. Their analysis indicated that chevrons had a minimal effect on vehicle speeds, with drivers adjusting back to their previous speeds as they became accustomed to the treatment.

The advisory speed sign is also a widely used speed-control measure. Kwon et al. proposed a two-stage speed reduction scheme using variable advisory speed limits at work zones [35]. The field data indicated a 25 to 35 percent reduction of the average one-minute maximum speed difference along the work zone area after the system was implemented. Hou et al. found that advisory speed limit signs were partially effective in 77 percent of the states while 15 percent found to be ineffective [36]. In addition, staggered speed limit reduction has been used by 48 percent of the states and found to be partially effective. Voigt et al. proposed a dual-advisory speed signing scheme that provided different recommended advisory speeds for trucks and passenger vehicles, in order to comfortably and safely traverse freeway connector ramps [37]. Based on the results of their analysis of the average and the 85^th^ percentile speeds at the midpoint of each studied curve, the dual-advisory warning signs were generally found to have a positive impact on reducing speeds at the point of the curvature and/or an accompanying reduction in speed-related crashes at the studied sites. Other studies have shown the value of using advisory speed signs in place of speed limit signs for transient geometric features, even freeway ramps [38].

The MUTCD recommends to use an advisory exit speed sign (AESS) where an engineering study shows it necessary to display a speed reduction message for ramp signage [16], a recommendation which was also adopted in the National Standard for Road Traffic Signs and Markings in China (code: GB5768-2009) [39]. An AESS installed along the deceleration lane may inform drivers the ideal driving speed in the deceleration lane, in order to avoid dramatic deceleration at the physical gore. The above two guidance documents also provide criteria for the application of an AESS under different circumstances. However, some details which are critical to the use of an AESS in engineering practice are still not to be specified. For example, what speed should be designated on the AESS, and to what degree can an AESS reduce the speeding and speed variance? These questions remain unanswered in the existing related studies.

In China, the speed limit is commonly 120km/h for the freeway mainline and 40km/h for exit ramps. Although an AESS is recommended by national standards, a single ramp speed limit sign is deployed around the physical gore for most freeway exit ramps in engineering applications. It is a common observation that drivers are not law-abiding based on a single speed limit sign. When departing the freeway mainline, drivers often maintain their freeway traveling speed in the deceleration lane and then decelerate dramatically around the physical gore, where the speeds are even higher than the posted speed limit. Such behaviors may lead to higher speed variances and potentiality more crashes [40,41]. Therefore, the primary objective of the study presented in this paper was to evaluate the effectiveness of the AESS in speed reduction at freeway exit ramps.

## Methodology

### AESS value

The advisory speed displayed should be based on a reliable engineering study [16]. The established engineering practices appropriate for the determination of the recommended advisory speed for a horizontal curve are as follows: design speed equation, traditional ball-bank indicator, and accelerometer. In China, no similar reference documents are available, even the posted ramp speed limit is usually determined by a speed measurement (such as 85% speed) or the design speed directly. Thus, a simple method was presented to calculate the AESS value before deploying it.

Calvi et al. [26] demonstrated that diverging drivers begin to decelerate before arriving at the deceleration lane, which disrupts the main flow; and the speeds recorded at the end of the deceleration lane usually exceed the design speed of the ramps. Moreover, drivers continue to decelerate on the ramp that follows the deceleration lane, and exiting drivers adopt speeds significantly lower than the speed of through traffic at the beginning of the deceleration lane. Therefore, the deceleration procedure of exiting vehicles was divided into four stages:

1) Vehicles decelerate from *V*_*T*_ on the freeway mainline to *V*_*t*_ at the starting point of the taper with a distance *L*_*1*_. 2) Vehicles decelerate from *V*_*t*_ at the starting point of the taper to *V*^*’*^_*t*_ at the theoretical gore with a rate *a*_*02*_ and a distance *L*_*2*_. 3) Vehicles decelerate on the deceleration lane from *V*^*’*^_*t*_ at the theoretical gore to *V*_*g0*_ at the physical gore with a rate *a*_*03*_ and a distance *L*_*3*_. 4)Vehicles decelerate on the curved ramp from *V*_*g0*_ at the physical gore to *V*_*r*_ at the section with the minimum radius of ramp with a rate *a*_*04*_ and a distance *L*_*4*_.

**Fig 1:**
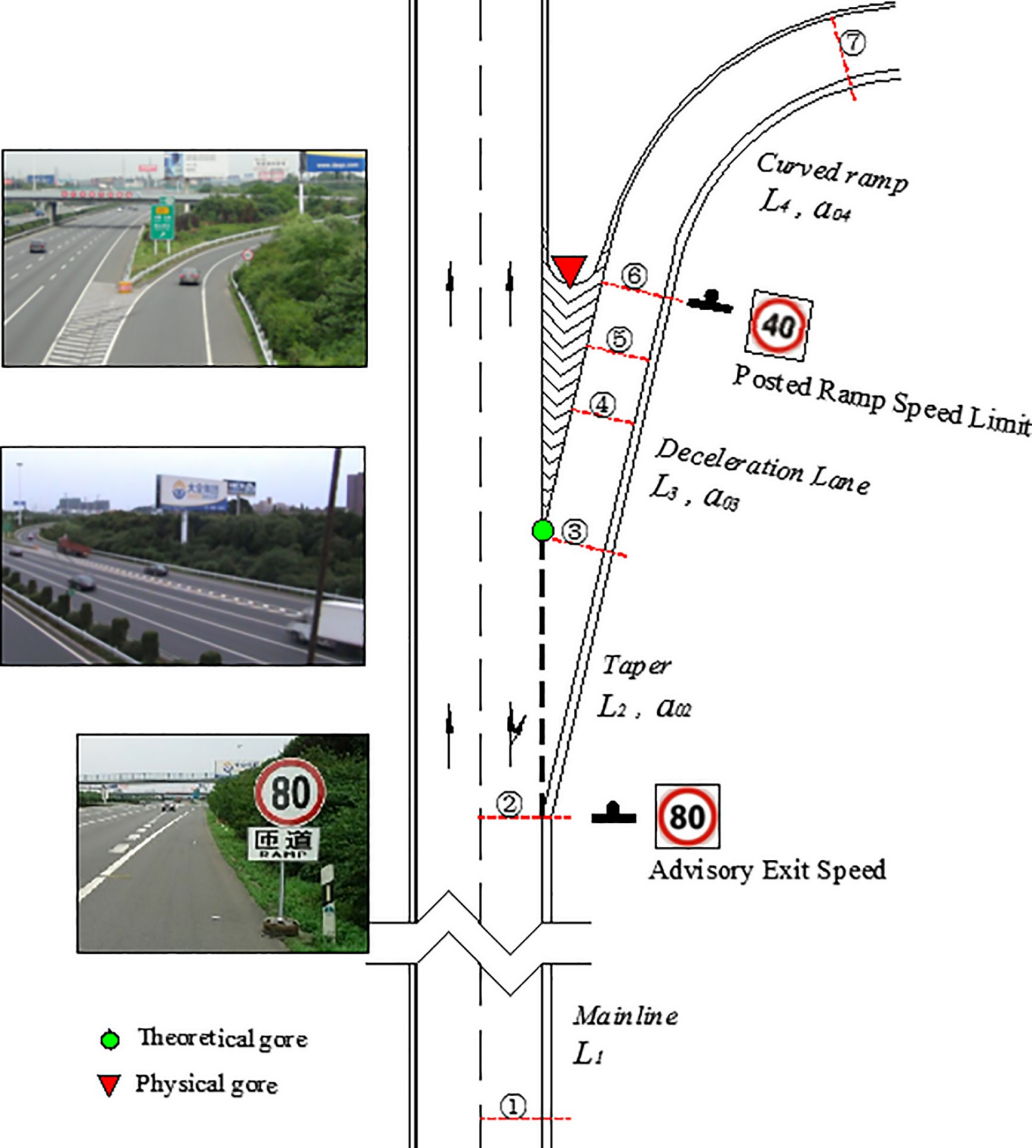
Layout of field data collection.

All vehicles are assumed to decelerate following the above procedure and can reduce to V_*r*_ at the section with the minimum radius of the ramp, which is also the posted ramp speed limit. The four stages of the deceleration process are illustrated in Fig 1. Theoretically, the procedure can be presented with the velocity-acceleration formula, as shown in Eq (1). If vehicles are assumed to be running at the limited speed at the minimum radius of the ramp, the running speed at the various upstream sections can be derived. Based on Eq (1), the theoretical running speed at the starting point of the taper can be derived using Eq (2).

$$V_{i}^{2}-V_{i+1}{}^{2}=2L_{i‐i+1}a_{i‐i+1}$$ (1)

$$\begin{array}{l} V_{t}=\sqrt{2a_{02}L_{2}+V_{t}^{'2}}\\ \;=\sqrt{2a_{02}L_{2}+2a_{03}L_{3}+V_{g0}^{2}}\\ \;=\sqrt{2a_{02}L_{2}+2a_{03}L_{3}+2a_{04}L_{4}+V_{r}{}^{2}} \end{array}$$ (2)

Where *a*_*i*-*i*+1_ is the deceleration rate between two neighboring sections, m/s^2^; *V*_*i*+1_ is the speed at section *i*+1, m/s; *V*_*i*_ is the speed at section *i*, m/s; *L*_*i*-*i*+1_ is the length between section *i* and section *i*+1, m; *i* is the code of the section; *V*_*r*_ is the posted speed limit, m/s; *V*_*g0*_ is the speed at the physical gore, m/s; *V*^*’*^_*t*_ is the speed at the theoretical gore, m/s; *V*_*t*_ is the speed at the starting point of the taper, m/s; *L*_*2*_ is the length of the taper, m; *L*_*3*_ is the length of the deceleration lane, m; *L*_*4*_ is the distance between the end of the deceleration lane and the section with the minimum radius of the ramp, m; *a*_*02*_ is the deceleration rate on segment *L*_*2*_, m/s^2^; *a*_*03*_ is the deceleration rate on segment *L*_*3*_, m/s^2^; and _*04*_ is the deceleration rate on segment *L*_*4*_, m/s^2^. The parameters a_*02*_, a_*03*_, a_*03*_, *L*_*2*_, *L*_*3*_, and *L*_*4*_ were obtained through a field study.

In practice, a speed limit cannot guarantee that all vehicles will drive under the posted speed. A revised model was introduced to improve the performance of the speed limit; and most of the past researchers examined the relationship between the operating speed and the posted speed limit. For example, Fitzpatrick [42] demonstrated the strong relationship between the two speeds in NCHRP Project 15–18, finding that no other roadway variable was statistically significant at a five percent alpha level, except for the posted speed limit. Wang et al. [42] also completed some related studies in China and proposed models based on Fitzpatrick’s model, as shown in Eq (3), which was presented to calculate the value of AESS $V_{g}^{'}$.

$$V_{g}^{'}=\frac{V_{t}-36.754}{0.497}$$ (3)

Where $V_{g}^{'}$ is the value of AESS, km/h, *V*_*t*_ is the speed at the starting point of the taper.

The final value of AESS *V*_*g*_ was determined with engineering experience based on the revised $V_{g}^{'}$. Arithmetic rounding, which is commonly used by engineers, was applied in this study. In practice, the sign value can also be determined in the field study, such as the 85% percentile speed

### Statistical method

A statistical method, the t-test, was used to test the differences between the speed data of two scenarios: “with AESS” and “without AESS.” The facility could be determined as effective only if the differences between the two conditions were significant. The null hypothesis presented in Eq (4) states that the two means are equal, and the assumption suggests that the sample sizes were large and approximate normality for the populations. The test statistic Z* is shown in Eq (5):

$$\begin{array}{l} H_{0}:\mu_{1}-\mu_{2}=0\\ H_{a}:\mu_{1}-\mu_{2}\neq 0 \end{array}$$ (4)

$$Z^{*}=\frac{\left(\bar{X_{1}}-\bar{X_{2}})-(\mu_{1}-\mu_{2}\right)}{\sqrt{\frac{s_{1}{}^{2}}{n_{1}}+\frac{s_{2}{}^{2}}{n_{2}}}}$$ (5)

Where H_0_(H_a_) is the null hypothesis (the alternative hypothesis), μ_1_(μ_2_) is the population mean of sample 1 (or sample 2), 95%, $\bar{X_{1}}(\bar{X_{2}})$ is the sample average, $s_{1}^{2}(s_{2}^{2})$ is the population variance of sample 1(or sample 2), and n_1_(n_2_) is the size of sample 1 (or sample 2).

The expression in the denominator is the standard error of the difference between the two sample means and requires two independent samples.

## Data collection

The field study were approved by the traffic management authority. In China, it is illegal to carry out any activities on a freeway without the permission of the traffic management authority. During the field test, the portable sign was deployed with the help of the traffic policeman who guaranteed the researchers’ safety issues. After that, both policeman and researchers leave the scene in order to avoid any disturbance to the drivers. Finally, the time of vehicles crossing each section is recorded by means of multi-point arrangement of radar and camera on the roadside, and the velocity of each section is obtained through post-processing. Note that the speed limit in this study has been discussed by traffic experts and traffic policeman with safety consideration. Since this study adopts the roadside data, which does not involve the collection of drivers’ data, it does not need the approval of the ethics committee.

There are various configurations of freeway exit ramps with different quantities and alignments of deceleration lanes. It is important to clarify the exit ramp configuration before undertaking a field study. Lu et al. [43] investigated 428 freeway exit ramps from ten megacities and major provinces in China and found that about 59 percent of them were tapered freeway exit ramps. The tapered exit ramp thus was taken as an example to measure the influences of an AESS in this study. During the site selection process, other influencing factors were also considered as follows: 1) the geometric design and other countermeasures, such as pavement, markings, etc., that can provide drivers with convenient and comfortable service; 2) no curves are ahead of freeway exit ramps, which can guarantee that all drivers can see and react to the AESS; 3) no other devices for speed management, such as transverse rumble strips, speed bumps, etc., were deployed around freeway exit ramp areas except for the posted freeway ramp speed limit; and 4) most vehicles are free-flowing, with no heavy traffic jams occuring around freeway exit ramps.

Three typical exit ramps were identified in Jiangsu, China. Site 1 was located in a rural area, where the ramp was part of a system interchange which connects to another freeway mainline; Site 2 was located in a suburban area, and the exit ramp connects to a toll station, for which drivers must stop or proceed at a slow speed; and Site 3 was an exit ramp connected to a service area where drivers stop and rest. The functions of the three ramps are different, which could obviously affect driving behaviors and speed distributions. The geometric and traffic characteristics of the sites are summarized in Table 1.

**Table 1:** Characteristics of sample sites.

Parameters		Site 1	Site 2	Site 3
		Ningxuan Freeway	Huning Freeway	Ninghang Freeway
Posted speed limit on mainline (km/h)		120	120	120
Posted ramp speed limit *V*_*g0*_ (km/h)		40	40	40
Number of lanes on on-way mainline		2	4	2
Lane width (m)	mainline	3.75	3.75	3.75
	exit, ramp	4.0	4.0	4.0
Length (m)	*L*_*1*_	200	200	200
	*L*_*2*_	135	120	120
	*L*_*3*_	65	120	80
	*L*_*4*_	80	90	50
Ramp radius (m)		120	130	80
Average deceleration rate (m/s^2^)	*a*_*02*_	0.263	0.277	0.452
	*a*_*03*_	1.122	1.051	1.316
	*a*_*04*_	0.829	0.419	0.203
Speed (km/h)	*V*_*t*_	78.3	81.9	77.4
	*V*_*g*_^*’*^	83.5	90.8	81.9
	*V*_*g*_	80	90	80

In order to obtain a more detailed speed distribution, seven sections were selected for conducting the field study. Section 1: 200m ahead of the starting point of the taper; Section 2: the starting point of the taper, where most exiting vehicles drive out of the mainline; Section 3: the theoretical gore, where exiting vehicles drive to the deceleration lane; Section 4: the midpoint of the deceleration lane; Section 5: the quarter of the deceleration lane near the physical gore; Section 6: the physical gore, where the vehicles enter the curved ramp; and Section 7: the section with the minimum radius of ramp, where the speed might be minimal.

In the Highway Capacity Manual [44], the recommended length of the diverging influence area is 1,500 ft (457m), which is defined from the point where the edges of the travel lanes of the merging roadways meet to a point 1,500 ft (457m) upstream of that point, i.e., the sum of *L*_1_, *L*_2_ and *L*_3_. Furthermore, A.Calvi etc. demonstrated that diverging drivers begin to decelerate before arriving at the deceleration lane, and the decision point is approximately 200m ahead of the starting point of taper[10]. However, similar recommended values are absent in China. The purpose of the field study was to measure vehicle speeds on the mainline, which should not be affected by the following exit. Considering the sum of *L*_2_ and *L*_3_ are 200m (site 1 and site 3) and 240m (site 2) respectively, *L*_*1*_ was identified as 200 meters in length.

The two scenarios, scenario 1 without AESS and scenario 2 with AESS, were developed at each site. Twelve hours (9:00 am to 11:00 am for each scenario, at each site, on two successive weekdays) were dedicated to data collection. In this study, Tuesdays and Wednesdays with good weather of two weeks were selected to collect data of two scenarios. For scenario 1 and scenario 2, there were 38 and 41 vehicles at Site 1, and 153 and 127 vehicles at site 2, while at site 3, 63 and 58 vehicles were recorded. Two methods (radar guns and video cameras) were used to collect the speed and deceleration data. Specifically, for sections 2 through 6, the speed profiles were obtained from videos; for the other two sections, radar guns were used to collect the data. The field study layout is shown in Fig 1. A portable AESS was deployed with the help of the police who guaranteed the researcher’s safety. When collecting data, the observers were careful to conceal themselves properly, to avoid influencing drivers’ decisions. If one or more vehicles were following a vehicle, the following vehicles’ speeds generally were heavily affected by the first vehicle. Therefore, to analyze the influences of the AESS, the speed and deceleration rate were collected only for the first vehicle under the aforementioned conditions.

During the analysis of the field data, a significant variance of speed at section 1 was seen between the two scenarios at sites 1 and 3, which was abnormal. After double checking the experimental scheme, we found that different observers had been arranged to record the field speed data using radar guns in the two scenarios. The speed variance may have been caused by the different operating habits of the observers. Consequently, the data collected at section 1 were discarded due to this inconsistency.

## Results and analysis

### Speed data

**Fig 2:**
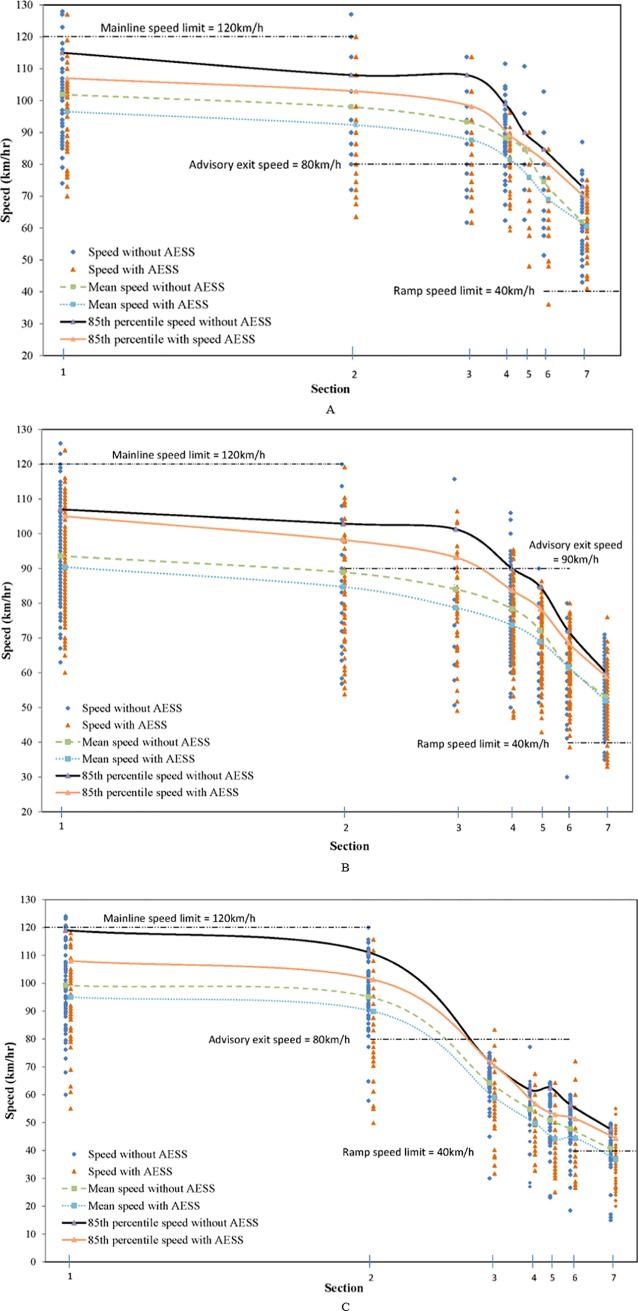
Speed profiles at ramp area. (A) Site 1. (B) Site 2. (C) Site 3.

Table 2 summarizes the changes in mean speed, the 85^th^ percentile speed, coefficient of variation, and percentages of vehicles exceeding the posted speed. To illustrate the data concisely, four critical sections 2, 3, 6 and 7 were selected. The speed profiles for all sections are illustrated in Fig 2.

**Table 2:** Speeds statistic before and after installation of AESS.

		Section 1		Section 2		Section 3		Section 4		Section 5		Section 6		Section 7	
		*V*_*1*_^a^	*V*_*1*_^*’*^^b^	*V*_*2*_	*V*_*2*_^*’*^	*V*_*3*_	*V*_*3*_^*’*^	*V*_*4*_	*V*_*4*_^*’*^	*V*_*5*_	*V*_*5*_^*’*^	*V*_*6*_	*V*_*6*_^*’*^	*V*_*7*_	*V*_*7*_^*’*^
**Site 1**	Min	74.0	70.0	72.0	63.5	61.7	61.7	62.4	59.2	62.6	48.0	51.4	36.0	43.0	41.0
	Mean	101.9	96.5	98.0	92.3	93.3	87.6	88.3	81.4	84.6	76.0	74.5	69.0	61.9	60.6
	Max	128.0	127.0	127.1	120.0	113.7	113.7	111.6	97.2	110.8	90.0	102.9	84.7	87.0	75.0
	V_85_^c^	115.0	107.0	108.0	102.9	108.0	98.2	98.9	89.0	90.0	84.7	84.7	80.0	73.0	69.0
	Std.	13.2	13.1	13.2	13.1	12.7	11.6	10.6	9.2	9.7	9.7	10.1	9.7	10.2	8.1
	ΔV_mean_^d^	5.4		5.7		5.7		6.9		8.6		5.5		1.3	
	ΔV_85_^e^	8.0		5.1		9.8		9.9		5.3		4.7		4.0	
	*P*^f^	0.075		0.059		0.042		0.03		0.000		0.016		0.530	
**Site 2**	Min	63.0	60.0	56.0	53.8	50.6	49.0	50.0	47.1	51.4	42.9	30.0	38.6	35.0	33.0
	Mean	93.6	90.4	88.9	84.7	84.0	78.7	78.4	73.7	72.2	69.0	61.5	61.7	53.1	51.9
	Max	126.0	124.0	120.0	119.2	115.7	106.4	106.0	95.4	90.0	86.3	80.0	80.0	71.0	76.0
	V_85_	107.0	105.0	102.9	98.2	101.3	93.2	90.0	83.7	84.7	78.4	72.0	68.6	60.0	59.0
	Std.	12.1	12.4	12.3	13.7	11.6	12.6	10.0	10.4	9.1	9.0	8.3	7.8	7.2	7.6
	ΔV_mean_	3.2		4.2		5.3		4.7		3.2		-0.2		1.2	
	ΔV_85_	2.0		4.7		8.1		6.3		6.3		3.4		1.0	
	*P*	0.031		0.007		0.000		0.000		0.003		0.822		0.183	
**Site 3**	Min	60.0	55.0	57.9	49.8	30.0	31.7	27.0	32.7	23.1	25.0	18.5	26.7	15.0	20.0
	Mean	99.2	95.0	95.2	89.9	64.3	58.9	54.8	49.8	50.9	44.1	47.7	44.4	40.6	36.9
	Max	124.0	118.0	120.0	115.7	75.0	83.3	77.1	67.5	64.3	64.3	60.0	72.0	49.7	55.0
	V_85_	119.0	108.0	111.1	101.3	71.9	70.3	61.8	56.8	62.5	52.9	56.4	51.4	47.6	44.4
	Std.	15.8	14.1	13.8	15.6	7.6	10.4	8.0	8.3	9.9	8.6	8.9	9.6	7.5	7.5
	ΔV_mean_	4.2		5.3		5.4		5.0		6.8		3.3		3.7	
	ΔV_85_	11		9.8		1.6		5.0		9.6		5.0		3.2	
	*p*	0.127		0.051		0.001		0.001		0.000		0.053		0.108	

^a^ Speed of section i without advisory exit speed limit.

^b^ Speed of section i with advisory exit speed limit.

^c^ The 85th percentile speed.

^d^ Difference in mean speed of *V*_*i*_ and *V*_*i*_^*’*^.

^e^ Difference in 85th percentile speed of *V*_*i*_ and *V*_*i*_^*’*^.

^f^ Probability value of t-Test.

Site 1: The results of the t-tests showed that the mean speeds measured at sections 2 through 6 with the AESS were significantly lower than those measured without the AESS (p<0.05). The reduction in the 85th percentile speed was also significant, varying from 4.0 km/h to 9.9 km/h. Furthermore, the percentage of speeding vehicles at sections 2 and 3 declined significantly. However, at section 7, the mean speed differences between the two scenarios were not found to be statistically significant. Although the percentage of speeding vehicles remained unchanged, the speed C.V. declined from 0.14 to 0.12, which indicated that the speed distribution tended to be more contracted after the AESS was installed. Site 2: At sections 2 through 5, the reductions in the mean speed and 85th percentile speed were significant after the AESS was installed. However, the AESS was ineffective at the last two sections, where the mean speed reduction was not significant. Furthermore, the percentage of speeding vehicles changed minimally at all the sections after the AESS was installed. Site 3: The significant reduction in the mean speed and 85th percentile speed occurred at all the sections, varying from 3.3 km/h to 5.3 km/h and from 1.6km/h to 9.8km/h respectively, especially around the physical gore. The percentage of speeding vehicles declined with the increased speed C.V. at all the sections after the AESS was installed, which indicated that the speed distribution tended to be more discrete.

In general, after installing the AESS, the mean deceleration rates in the taper and deceleration lane increased slightly. The t-test results for the mean speed indicated significant speed reductions in sections 2 through 5 (the taper and deceleration lane) at all three sites, which proved that drivers were taking earlier deceleration maneuvers in the taper and the deceleration lane after the AESS was installed. Furthermore, the percentage of speeding vehicles also decreased at site 1 and site 3, substantiating the effectiveness of the AESS as a speed control countermeasure. However, for the curved ramps at site 2 and 3, the mean speeds were not significantly reduced, and only a few vehicles were observed as driving under the posted ramp speed limit, which indicated that the AESS had no significant positive effects at the curved ramp.

Although most drivers decelerated significantly in advance as a response to the AESS, the duration time could have been affected by other exit ramp factors, including the length of the deceleration lane and taper, the purpose of the exit ramp, and the drivers’ characteristics, which could have affected their driving behaviors and resulted in the varying speed differences around the physical gore and curved ramp between the two scenarios. Firstly, the length of the deceleration lane at site 2 was 240 meters, which was longer than those at sites 1 and 3 (200 meters); drivers tend to be more cautious when driving in a shorter deceleration lane. Secondly, the ramp at site 1 was part of an interchange in a rural area and most of the drivers were non-commuters; therefore, it was not necessary for drivers to slow down dramatically, since the ramp was connected to another freeway mainline. The ramp at site 2 was located in a suburban area and connected to a toll station, where perhaps some drivers were commuters and familiar with the ramp configurations. After passing through the area with AESS where there might be a significant speed reduction, drivers would likely adjust their speed more smoothly, thereby resulting in the small difference at sections 6 and 7. The ramp at site 3 was connected to a service area, where most drivers perhaps were not in a hurry, and who followed the AESS and maintained a lower speed until section 7.

### Deceleration rate data

**Fig 3:**
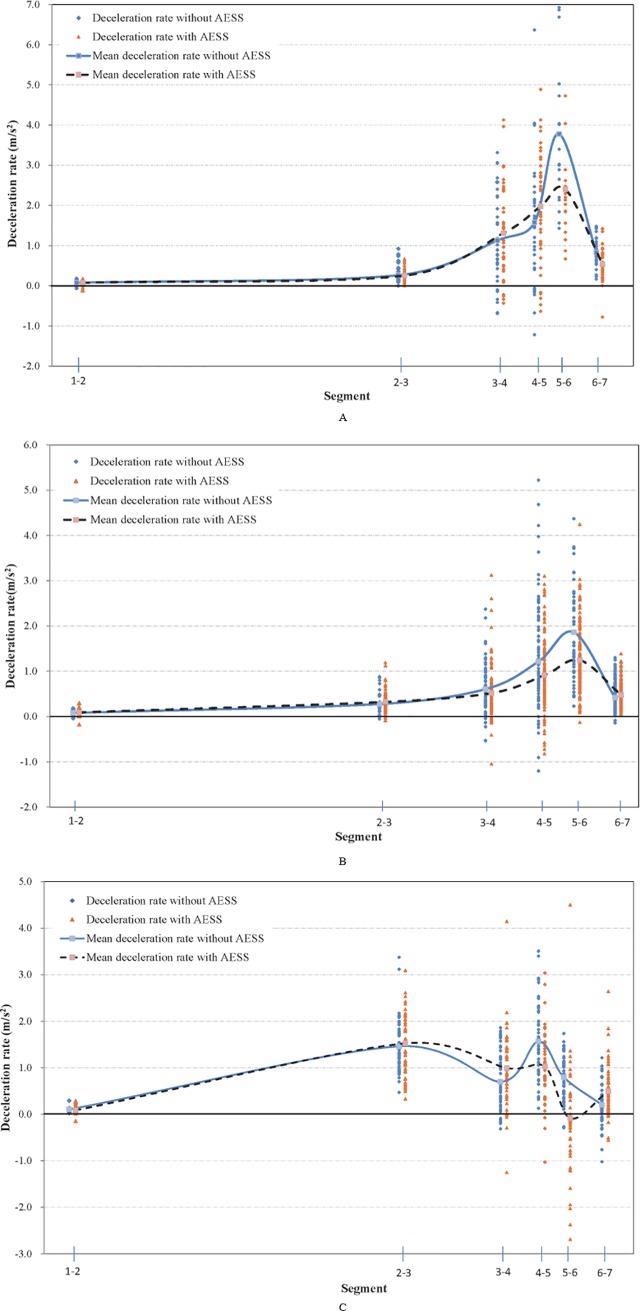
Deceleration rate profiles at three sites. (A) Site 1. (B) Site 2. (C) Site 3.

Summary statistics of the deceleration rates measured at the three sites for the two scenarios are shown in Table 3.The deceleration rate profiles are illustrated as well in Fig 3.

**Table 3:** Summary of statistics on deceleration rate at three sites.

		Section 1–2		Section 2–3		Section 3–4		Section 4–5		Section 5–6		Section 6–7	
		*a*_*1-2*_^a^	*a*_*1-2*_^*’*^^b^	*a*_*2-3*_	*a*_*2-3*_^*’*^	*a*_*3-4*_	*a*_*3-4*_^*’*^	*a*_*4-5*_	*a*_*4-5*_^*’*^	*a*_*5-6*_	*a*_*5-6*_^*’*^	*a*_*6-7*_	*a*_*6-7*_^*’*^
**Site 1**	Min	-0.062	-0.113	0.000	0.000	-0.690	-0.436	-2.037	-0.636	1.429	0.670	0.164	-0.781
	Mean	0.075	0.076	0.263	0.254	1.128	1.310	1.575	1.975	3.775	2.401	0.829	0.542
	Max	0.173	0.175	0.920	0.670	3.312	4.127	8.496	4.886	6.923	4.727	1.475	1.423
	Std.	0.049	0.052	0.237	0.203	1.103	1.113	1.957	1.365	1.734	1.076	0.338	0.414
**Site 2**	Min	-0.037	-0.176	-0.052	-0.088	-0.537	-1.043	-1.201	-0.821	0.231	-0.126	-0.139	-.088
	Mean	0.082	0.150	0.277	0.340	0.607	0.503	1.217	0.830	1.860	1.107	0.419	0.469
	Max	0.181	0.988	0.880	1.191	2.373	3.125	5.222	3.099	4.370	4.246	1.303	1.391
	Std.	0.036	0.169	0.211	0.220	0.457	0.514	1.025	0.745	0.781	0.779	0.288	0.273
**Site 3**	Min	0.024	-0.146	0.470	0.331	-0.314	-1.252	0.129	-1.027	-0.290	-2.689	-1.022	-0.556
	Mean	0.104	0.086	1.460	1.524	0.698	0.991	1.564	1.025	0.796	-0.083	0.203	0.498
	Max	0.291	0.285	3.374	3.090	1.862	4.147	3.508	3.038	1.736	4.504	1.215	2.639
	Std.	0.032	0.063	0.571	0.649	0.615	0.760	0.793	0.749	0.471	1.120	0.397	0.544

^a^ Deceleration rate between Section i and Section i+1 without AESS (m/s^2^).

^b^ Deceleration rate between Section i and Section i+1 with AESS (m/s^2^).

For the three sites, the vehicles decelerated from the first section at a smaller rate of deceleration rate and then usually reduced their speeds greatly at section 2 or section 3, which varied with the type of exit ramp. At Site 2, the smaller deceleration rate lasted from section 1 to section 3. When vehicles passed the theoretical gore, drivers began to decelerate by applying pressure on the brake pedal sharply, which resulted in a greater deceleration rate. In contrast, drivers released their feet from the acceleration pedal between the first section to the starting point of the taper (section 2) and continued to decelerate with the brake between the taper and the physical gore at Site 3. The length of the deceleration lane and the purpose of the exit ramp could have contributed to the difference for the two sites. The length of the deceleration lane at Site 2 was 240 meters, which was longer than that of Site 3 (200 meters); and drivers tend to be more cautious when driving in a shorter deceleration lane. As shown in Table 2, the speeds at Site 1 were generally much greater than those at the other two sites, and the continuous flow characteristics could explain it as well. At Site 1, drivers did not need not stop their cars to drive through the exit since the ramp was connected to another freeway mainline. The situation at Site 2 and Site 3 was different as drivers were required to stop at the service area or toll station, which could obviously affect their driving behaviors.

**Fig 4:**
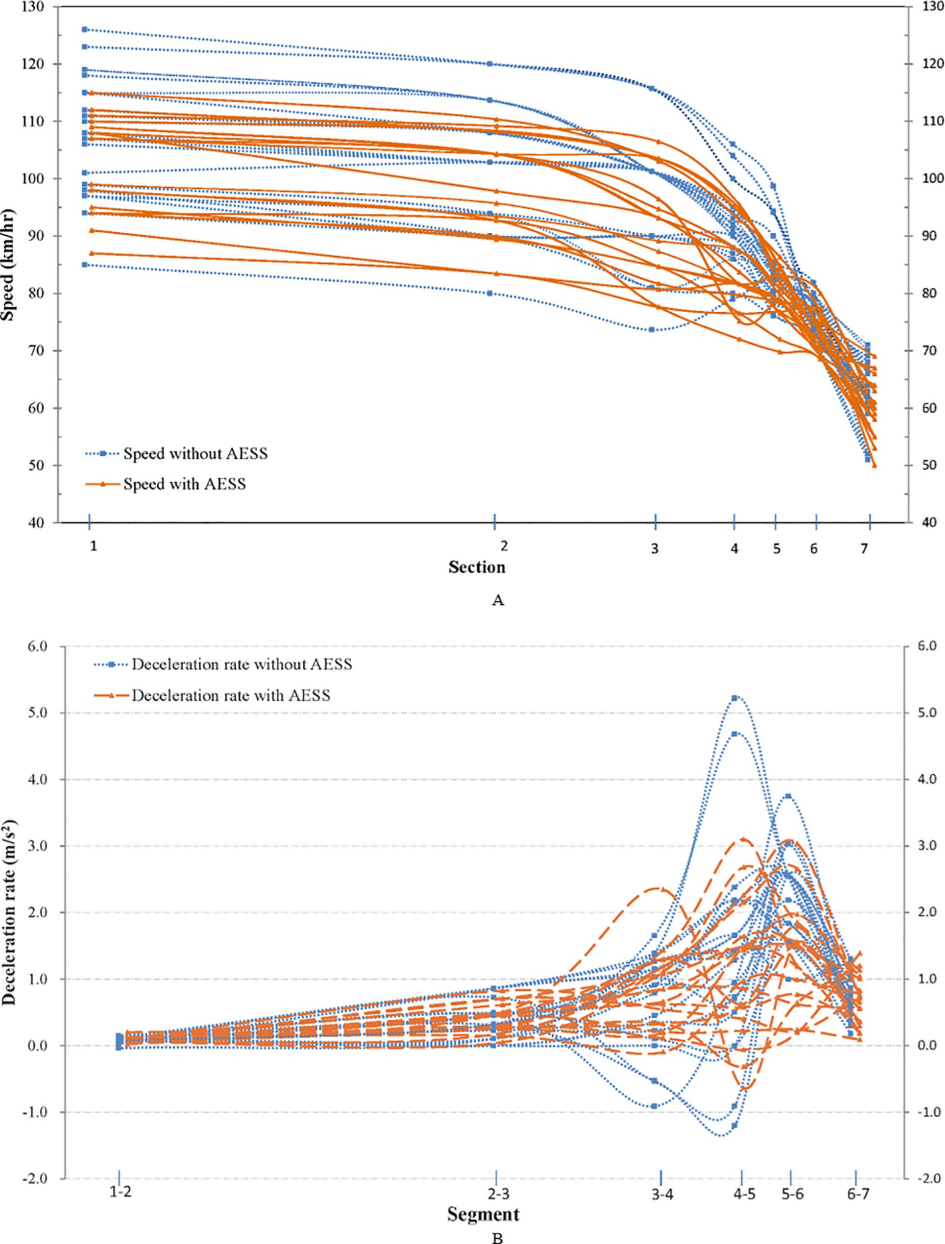
Profiles of speed and deceleration rates over the 85th percentile at physical gore of Site 2. (A) Speed Trajectory.(B) Deceleration Rate Trajectory.

The two mean deceleration rate curves, as shown in Fig 4, revealed that the curve with the AESS was smoother around the physical gore, which could contribute to a potential reduction in rear-end crashes; and some of the large deceleration rates improved around the physical gore. In section 1 through section 3 of Site 3, there was no significant difference between the two scenarios, and the deceleration rates were negligible, which means their influence on the mainline vehicles could be minimal. In section 3 through section 5, a significant increase occurred, and the deceleration rates in scenario 2 were larger, which showed that drivers were taking earlier actions in the deceleration lane after the AESS was installed. With the earlier deceleration action, drivers decelerated slightly at the last two sections. Similar finding also were derived for Site 2. At Site 3, the significant changes in the deceleration lane and around the physical gore also occurred with the AESS, but the changes were not as significant as the other two sites. However, it was clear that sufficient deceleration was achieved ahead of the physical gore with the AESS; and it was not necessary to decelerate greatly around the physical gore, which also was the case in scenario 1. Interestingly, in section 5 and section 6, many vehicles accelerated slightly around the physical gore with the AESS, which might have resulted from sufficient deceleration in the deceleration lane and a sloping and short ramp at Site 3.

After installing the AESS, the mean deceleration rates in the deceleration lane increased slightly, which could reduce the opportunity of speeding at the physical gore. The mean deceleration rates declined at the sections around the physical gore, which might have resulted from sufficient deceleration ahead of the physical gore and thus showed the effectiveness of the AESS in that the dramatic speed reduction in the deceleration lane was improved.

### Speed and deceleration rate over the 85th percentile

Physical gore plays an important role in a freeway exit ramp system. Ramp-related motor vehicle crashes occur most often at this particular point [10]. Therefore, vehicles whose speeds exceeded the 85th percentile at the physical gore of site 2 were used as an example to further analyze the effectiveness of the AESS. From the perspective of traffic safety, these vehicles were at high risk of a crash, if they were not able to decelerate to an appropriate lower speed in a limited time at the curved ramp. The trajectories of the speed and deceleration rates are illustrated in Fig 4. The total number of vehicles was 23 and 19 for scenario 1 and scenario 2, respectively.

Even though a majority of these vehicles were still speeding in the deceleration lane and on the ramp, they decreased their speed after the AESS was installed. Meanwhile, the ranges of speed were reduced (42.1 km/h in scenario 1 and 28.8 km/h in scenario 2 at section 3), which indicated that the vehicles running at higher speeds were influenced by the AESS significantly. Consequently, the speed variance was reduced effectively, especially in the taper and deceleration lane (sections 2 through 5). As far as the deceleration rate profiles, significant differences occurred in sections 3 through 7. More drivers decelerated in advance when the AESS was installed, which allowed them a smaller deceleration rate around the physical gore. This tendency could lead to reductions in speed and, consequently, the potential number of crashes. Although a few adventurous drivers accelerated and decelerated sharply in both scenarios, the fluctuation range of the deceleration rate at the last four sections with the AESS was much smaller than those without the AESS. In sum, most drivers were able to slow down at a relatively continuous and stable deceleration rate after the AESS was installed, which proved that the AESS had a potentially positive effect on reducing speeding.

## Conclusions

This study sought to evaluate the effectiveness of the AESS in speed reduction at freeway exit ramps. Three sites with similar exit ramp configurations were selected and two scenarios (with AESS/without AESS) were designed to quantify the influences of the AESS on the speed of exiting vehicles. The presence of AESS had different effects on the three typical ramps. Generally speaking, the AESS was effective in reducing the mean speed and 85^th^ percentile speed, especially in the taper and deceleration lane. Drivers tended to decelerate in advance when the AESS was installed, which led to a smooth deceleration process, especially on the segment between the theoretical gore and the physical gore. Furthermore, the AESS was also helpful in reducing aggressive driving behaviors to some extent. Although the effects of the AESS on speed reduction at curved ramps were not ideal, the speed fluctuation range tended to be more contracted when the AESS was installed.

Many factors contribute to ramp crashes, including driver impairment, fatigue, visual deficits, and speeding. In China, speeding is a common occurrence in exit ramp areas. Thus, for some countermeasures, even seemingly small reductions in speed can likely result in significant safety benefits. The study presented in this paper was limited by the small number of experimental ramps and the short-term data collection, which resulted from the limited support of the police. However, even a small data sample can provide preliminary proof that the AESS can be effective in reducing speeds and speeding. The configurations of some ramps are commonly limited by land use and generally have a small ramp radius and short deceleration lane. For those ramps, the most effective method for improving safety is ramp realignment or reconstruction. However, such measures are not always feasible due to funding and right-of-way restrictions. As a special low-cost speed control countermeasure, the AESS could be implemented easily, which could improve traffic safety to some extent.

The location of the AESS can impact its effectiveness, especially in terms of the influence of the area on speed reduction. Further study on this issue can be valuable, for example, evaluating the effect of moving the AESS to the starting point of the deceleration lane. Moreover, some drivers change their speeds frequently and might accelerate in the deceleration lane, and then decelerate sharply around the physical gore. Aggressive driving may account for those uncommon decisions, so future studies conducted on the causation and influences of aggressive driving at exit ramp areas would be helpful. Furthermore, when the AESS is installed, some vehicles on the mainline may decelerate in advance, which affects the traffic operation and safety performance of the freeway mainline. examining the effect of advisory speed limit in reducing crashes through this speed control countermeasure and in maintaining a certain level of traffic efficiency is also recommended.

## Supporting information

S1 File Data for three sites. (XLSX)
